# The Relationship between Corvis ST Tonometry Measured Corneal Parameters and Intraocular Pressure, Corneal Thickness and Corneal Curvature

**DOI:** 10.1371/journal.pone.0140385

**Published:** 2015-10-20

**Authors:** Ryo Asaoka, Shunsuke Nakakura, Hitoshi Tabuchi, Hiroshi Murata, Yoshitaka Nakao, Noriko Ihara, Ulfah Rimayanti, Makoto Aihara, Yoshiaki Kiuchi

**Affiliations:** 1 Department of Ophthalmology, The University of Tokyo, Tokyo, Japan; 2 Department of Ophthalmology, Saneikai Tsukazaki Hospital, Himeji, Japan; 3 Department of Ophthalmology and Visual Science, Hiroshima University, Hiroshima Japan; 4 Faculty of Health Science, UIN Alauddin Makassar, Sulawesi Selatan, Indonesia; Duke University, UNITED STATES

## Abstract

The purpose of the study was to investigate the correlation between Corneal Visualization Scheimpflug Technology (Corvis ST tonometry: CST) parameters and various other ocular parameters, including intraocular pressure (IOP) with Goldmann applanation tonometry. IOP with Goldmann applanation tonometry (IOP-G), central corneal thickness (CCT), axial length (AL), corneal curvature, and CST parameters were measured in 94 eyes of 94 normal subjects. The relationship between ten CST parameters against age, gender, IOP-G, AL, CST-determined CCT and average corneal curvature was investigated using linear modeling. In addition, the relationship between IOP-G versus CST-determined CCT, AL, and other CST parameters was also investigated using linear modeling. Linear modeling showed that the CST measurement ‘*A time-1*’ is dependent on IOP-G, age, AL, and average corneal curvature; ‘*A length-1*’ depends on age and average corneal curvature; ‘*A velocity-1*’ depends on IOP-G and AL; ‘*A time-2*’ depends on IOP-G, age, and AL; ‘*A length-2*’ depends on CCT; ‘*A velocity-2*’ depends on IOP-G, age, AL, CCT, and average corneal curvature; ‘*peak distance*’ depends on gender; ‘*maximum deformation amplitude*’ depends on IOP-G, age, and AL. In the optimal model for IOP-G, *A time-1*, *A velocity-1*, and *highest concavity curvature*, but not CCT, were selected as the most important explanatory variables. In conclusion, many CST parameters were not significantly related to CCT, but IOP usually was a significant predictor, suggesting that an adjustment should be made to improve their usefulness for clinical investigations. It was also suggested CST parameters were more influential for IOP-G than CCT and average corneal curvature.

## Introduction

Glaucoma is the second leading cause of blindness in the world and approximately 60 million people are affected with the disease.[1] Glaucoma is characterized by progressive retinal ganglion cell loss which results in irreversible visual field (VF) damage. Elevated intraocular pressure (IOP) is an established risk factor of glaucoma, however, recent research has revealed other important factors related to the occurrence and also progression of the disease. Central corneal thickness (CCT) has been proposed as a factor related to the progression of glaucoma; one study suggested that a thin cornea is associated with an increased risk, [2] however, a more recent study purported that corneal hysteresis (CH) measured with the Ocular Response Analyzer (ORA, Reichert Ophthalmic Instruments, Depew, NY, USA) is a risk factor of glaucoma, but CCT is not,[3] and indeed results from a randomized controlled study confirmed this finding.[4]

Recently the development of the Corneal Visualization Scheimpflug Technology instrument (Corvis ST tonometry: CST; Oculus, Wetzlar, Germany), a new non-contact tonometry device integrated with an ultra-high-speed Scheimpflug camera, has enabled the direct visualization of corneal movement during the application of a rapid air-puff; as a result, a much larger number of biomechanical properties have become measurable.

To date, the relationship between CST-derived measurements and various other ocular parameters, such as CCT, corneal curvature and axial length (AL) has not been investigated in detail. There is a previous report which analyzed the relationship among CST parameters, but without GAT measured IOP and also corneal curvature. [5] Also previous studies suggested the possible relationship between the corneal deformation and IOP.[6–8] Again, it is clinically very important to analyze the effect of corneal biomechanical parameters on IOP measurement with GAT, because IOP is the only manageable parameter when treating patients with glaucoma and indeed clinicians are making treatment decision basing on IOP record, usually with GAT. Thus, the first purpose of the current study was to investigate the correlation between CST parameters, considering IOP measured with GAT. Furthermore, it has been widely reported that IOP measurements from Goldmann applanation tonometer can be influenced by corneal biomechanics, such as CCT[9–19] and corneal curvature.[13,20,21] Consequently, the second purpose of the study was to investigate the effect of CST-determined corneal biomechanical parameters on IOP readings by GAT.

## Methods

The study was approved by the Research Ethics Committee of the Graduate School of Medicine and Faculty of Medicine at The University of Tokyo (Tokyo, Japan), Saneikai Tsukazaki Hospital (Himeji, Japan), and Hiroshima University Hospital (Hiroshima, Japan), and it was registered with the University Hospital Medical Network (UMIN) clinical trials registry. The registration title was ‘Intercomparison analysis of factors obtained from corneal biomechanical parameters measurements having different measuring principles’ and the registration number was JPRN-UMIN000016623. Written consent was given by patients for their information to be stored in the hospital database and used for research. This study was performed according to the tenets of the Declaration of Helsinki.

### Subjects

Data from 94 normal eyes of 94 subjects were prospectively acquired at Saneikai Tsukazaki Hospital and Hiroshima University Hospital between February 2014 and December 2014. Exclusion criteria were abnormal eye-related findings except for clinically insignificant senile cataract on biomicroscopy, gonioscopy and funduscopy, and history of ocular diseases, such as glaucoma or age-related macular degeneration. Only the subjects age ≥ 20 years old were included. IOP was not used for exclusion in order that a wide range of IOPs were considered in the analysis; thus normal eyes were defined not using the IOP level and as a result, previously undiagnosed ocular hypertensive eyes can be included. None of the subjects were using eye drops, especially anti-IOP agents, which can change the biomechanical properties of the cornea.[22–25] Subjects with diabetes mellitus were not included, because of the possible effects of the disease on corneal hysteresis.[26–28]

### Corvis ST tonometer measurements

Measurements with the CST (Ver1.00r30) were carried out three times per patient and the average of all three measurements were used in statistical analyses. An approximately one minute interval was given between each measurement during when data storage and processing operations are carried out within the CST instrument.

The principles of the CST have been described in detail elsewhere.[29,30] Briefly, a high-speed Scheimpflug camera takes over 4,000 frames/second images to monitor corneal response to an air-puff pulse that forces the cornea inward until it reaches a concavity phase. A number of parameters are recorded: ‘*A time-1/-2*’ is the length of time from the initiation of the air puff to the first (when the cornea is moving inwards) or second applanation (when the cornea moves outwards); ‘*A length-1/-2*’ is the length of the flattened cornea at the first or second applanation; ‘*velocity-1/-2*’ is the corneal velocity during the first or second applanation; ‘*highest concavity time*’ is the length of time from the start of deformation to the point when the cornea reaches highest concavity; ‘*highest concavity curvature*’ is the central curvature radius at the highest concavity; ‘*peak distance*’ is the distance between the two surrounding peaks of the cornea at the highest concavity; ‘*maximum deformation amplitude'* is the movement of the corneal apex from the start of deformation to the highest concavity; the movement of the corneal apex is compensated by the movement of whole eye and hence, only the movement of the cornea is described by this parameter.

### Other ocular measurements

IOP measurements with Goldmann applanation tonometry (IOP-G) measurements were carried out, post-CST, three times after instillation of topical 0.5% tetracaine; the average value was used in statistical analyses. The tonometer was set at 10 mm Hg before each reading. AL and corneal curvature were measured using the IOL master (Carl Zeiss Meditec). The average value of the maximum and minimum corneal curvatures was used in analyses.

### Statistical analysis

The influence of measurement time (first, second or third) on measured CST parameters was investigated using the linear mixed model. The coefficient of variance (CV) and intraclass correlation (ICC) of the CST parameters were calculated. Subsequently, the relationship between *A time-1* and parameters: age, gender, IOP-G, AL, CST-determined CCT and average corneal curvature was analyzed using the linear mixed model, in which each subject was treated as a random effect; see Table 1. Similarly, the relationships between the various other CST parameters (*A time-2*, *A length-1/-2*, *velocity-1/-2*, *highest concavity time*, *highest concavity curvature*, *peak distance*, and *maximum deformation amplitude*) and the above six ocular/systemic parameters were analyzed using the simple linear regression model, as shown in Table 1. The optimal linear models were selected among all possible combinations of predictors: 2^6^ patterns (in investigating the relationship between CST parameters against age, gender, IOP-G, AL, CST-determined CCT and average corneal curvature) based on the second order bias corrected Akaike Information Criterion (AICc) index. The AIC is a common statistical measure used in model selection, and the AICc is a corrected version of the AIC, which gives an accurate estimation even when the sample size is small.[31] The degrees of freedom in a multivariate regression model decreases with a large number of variables. It is therefore recommended to use model selection methods to improve the model fit by removing redundant variables.[32,33]

**Table 1:** Objective and explanatory variables analyzed. CST: Corvis ST tonometer, CCT: central corneal thickness, AL: axial length, IOP-G: intraocular pressure measured with Goldmann tonometrer.

objective CST parameters	explanatory parameters		
	CST parameters	Ocular parameters	Systemic parameters
A time-1	CCT	AL	age
A length-1		IOP-G	gender
A velocity-1		average corneal curvature	
A time-2			
A length-2			
A velocity-2			
highest concavity time			
highest concavity curvature			
peak distance			
maximum deformation amplitude			

In addition, the relationship between IOP-G versus CST-determined CCT, AL, average corneal curvature, gender, age and the ten other CST parameters (*A time-1*, *A length-1*, *velocity-1*, *A time-2*, *A length-2*, *velocity-2*, *highest concavity time*, *highest concavity curvature*, *peak distance* and *maximum deformation amplitude*) was analyzed using the simple linear regression model. The analysis was repeated without all ten additional CST-parameters, and also without *A time-1* in particular.

All analyses were performed using the statistical programming language ‘R’ (R version 2.15.1; The Foundation for Statistical Computing, Vienna, Austria).

## Results

Characteristics of the study subjects are summarized in Table 2.

**Table 2:** Subjects’ demographics. AL: axial length, IOP-G: intraocular pressure measured with Goldmann tonometry.

	value
age, (mean ± sd) [range], years old	52.1±23.4 [21 to 91]
male / female	41 / 53
right / left	59 / 35
AL	24.6±1.7 [21.6 to 30.3]
IOP-G	16.0±3.7 [8 to 25]
average corneal curvature, (mean ± sd) [range], ms	7.7±0.6 [7.0 to8.4]

CST-derived parameters are summarized in Table 3. *IOP-C* measurements were consistent across the three recordings (p >0.05, linear mixed model). Similarly, CCT measurements were not significantly different. The *A time-1* measurement did vary across the three recordings with the second measured value having a lower value (p = 0.041); however, this phenomenon was not observed between the first and third recordings (p >0.05, linear mixed model). The CV and ICC of each CST parameter are shown in Table 3; particularly high ICC values were observed for *CCT*, *IOP-C*, *A time-1*, *A time-2* and *maximum deformation amplitude*.

**Table 3:** Comparison of Corvis ST tonometer measured variables in each measurement. CCT: central corneal thickness, sd: standard deviation, CV: coefficient of variance, ICC: intraclass correlation.

	Measurement			average	CV (%)	ICC
	1st	2nd	3rd			
CCT, (mean ± sd) [range], μm	547.5±33.1 [453 to 643]	547.2±32.7 [458 to 645]	547.9±32.5 [453.0 to 643.0]	547.5±32.3 [454.7 to 643.7]	0.9±0.9 [0.0 to 5.1]	0.99
IOP-C, (mean ± sd) [range], mmHg	13.9±3.0 [5.0 to 23.0]	13.9±3.0 [4.5 to 19.5]	13.8±3.4 [4.0 to 22.0]	13.8±3.0 [4.5 to 20.5]	6.1±5.4 [0.0 to 30.0]	0.97
A time-1, (mean ± sd) [range], ms	7.3±0.3 [6.6 to 8.4]	7.3±0.3 [6.5 to 8.0]	7.3±0.3 [6.5 to 8.3]	7.3±0.3 [6.5 to 8.1]	1.2±1.0 [0.2 to 7.0]	0.95
A length-1, (mean ± sd) [range], mm	1.8±0.3 [1.3 to 2.3]	1.8±0.3 [1.3 to 2.4]	1.8±0.3 [1.2 to 2.4]	1.8±0.2 [1.4 to 2.3]	13.7±7.1 [1.0 to 30.4]	0.13
A velocity-1, (mean ± sd) [range], m/s	0.15±0.03 [0.05 to 0.23]	0.15±0.03 [0.09 to 0.24]	0.15±0.03 [0.07 to 0.22]	0.15±0.02 [0.09 to 0.21]	13.9±9.3 [0.0 to 47.6]	0.39
A time-2, (mean ± sd) [range], ms	21.9±0.4 [20.1 to 23.0]	21.9±0.4 [20.7 to 22.8]	21.8±0.7 [17.8 to 23.3]	21.9±0.4 [20.4 to 23.0]	0.9±1.6 [0.1 to 12.4]	0.75
A length-2, (mean ± sd) [range], mm	1.9±0.5 [1.0 to 2.9]	1.8±0.5 [0.9 to 2.7]	1.9±0.5 [0.6 to 2.7]	1.8±0.3 [1.0 to 2.7]	24.2±9.8 [3.4 to 52.8]	0.18
A velocity-2, (mean ± sd) [range], m/s	0.38±0.07 [-0.23 to -0.56]	0.39±0.09 [-0.17 to -0.71]	0.38±0.09 [-0.68 to 0.07]	0.39±0.07 [0.20 to 0.60]	12.7±12.6 [1.4 to 111.9]	0.75
highest concavity time, (mean ± sd) [range], ms	16.8±0.6 [15.3 to 18.3]	16.8±0.4 [15.7 to 17.8]	16.8±0.5 [14.1 to 18.3]	16.8±0.4 [15.6 to 18.0]	1.9±1.5 [0.0 to 10.4]	0.66
highest concavity curvature, (mean ± sd) [range], mm	7.0±0.8 [5.5 to 9.7]	7.0±1.1 [1.7 to 9.5]	6.9±0.9 [1.7 to 8.8]	7.0±0.7 [4.8 to 8.7]	8.1±8.7 [0.5 to 59.0]	0.68
peak distance, (mean ± sd) [range], mm	3.9±1.2 [2.1 to 5.4]	4.0±1.2 [2.3 to 5.6]	3.9±1.3 [2.2 to 5.6]	8.7±0.8 [2.3 to 5.5]	24.3±18.8 [0.1 to 49.9]	0.37
maximum deformation amplitude, (mean ± sd) [range], mm	1.1±0.1 [0.9 to 1.3]	1.1±0.1 [0.9 to 1.3]	1.1±0.1 [0.8 to 1.4]	5.5±1.1 [0.9 to 1.3]	3.2±1.9 [0.0 to 11.0]	0.95

Table 4 shows the explanatory parameters selected in the optimal linear model for the various CST parameters. In the optimal model for *A time-1*, IOP-G, age, AL, and average corneal curvature were selected among the six possible explanatory parameters (IOP-G, age, gender, AL, CST-determined CCT and average corneal curvature). For the *A length-1* model, age and average corneal curvature were selected. For *A velocity-1*, IOP-G and AL were selected. IOP-G, age, and AL were selected to explain *A time-2*. Only CCT was selected to describe *A length-2*. For *A velocity-2*, IOP-G, age, AL, CCT and average corneal curvature were selected. For *highest concavity time*, only AL was selected. For *highest concavity curvature*, IOP-G, age, AL, CCT, and average corneal curvature were selected. Gender was selected for a model of *peak distance*. IOP-G, age, and AL were selected to explain *maximum deformation amplitude*.

**Table 4:** Parameters selected in optimal linear models for each Corvis ST tonometer measured variable. CST: Corvis ST tonometer, AL: axial length, CCT: central corneal thickness, IOP-G: intraocular pressure measured with Goldmann tonometer. Numbers beneath selected variables represent the coefficients of the selected parameters (linear model).

CST parameter	selected parameters					
A time-1	IOP-G	age	AL		average corneal curvature	
	0.052	-0.0015	0.024		-0.15	
A length-1		age			average corneal curvature	
		0.0028			0.14	
A velocity-1	IOP-G		AL			
	-0.0017		-0.0016			
A time-2	IOP-G	age	AL			
	-0.045	-0.0037	-0.046			
A length-2				CCT		
				0.0029		
A velocity-2	IOP-G	age	AL	CCT	average corneal curvature	
	0.0077	0.00044	-0.014	0.00043	0.053	
highest concavity time			AL			
			-0.040			
highest concavity curvature	IOP-G	age	AL	CCT	average corneal curvature	
	0.038	0.0090	-0.14	0.0084	0.80	
peak distance						sex (male)
						-0.28
maximum deformation amplitude	IOP-G	age	AL			
	-0.014	0.0016	0.010			

As shown in the Table 5, CCT and average corneal curvature were selected in the optimal model for IOP-G, among age, gender, AL, CST-determined CCT, and average corneal curvature. However, when CST parameters were included as possible explanatory variables, *A time-1*, *A velocity-1*, and *highest concavity curvature* were selected in the optimal model for IOP-G; age, gender, AL, CCT, average corneal curvature, *A length-1*, *A time-2*, *A length-2*, *A velocity-2*, *highest concave time*, *peak distance* and *maximum deformation amplitude* were not selected. Excluding *A time-1* resulted in a different selection of CST parameters: AL, *A velocity-2*, *highest concavity time*, *maximum deformation amplitude* and average corneal curvature; CCT was not selected.

**Table 5:** Parameters selected in optimal linear models for IOP measured with Goldmann tonometer. CST: Corvis ST tonometer, IOP-C: intraocular pressure measured with Corvis ST tonometer, AL: axial length, CCT: central corneal thickness. Characters in bold suggest selected parameters. ‘n.s.’ suggests not selected in the optimal linear model.

without CST parameters		with CST parameters		with CST parameters (A time-1 excluded)	
parameter	coefficients	parameter	coefficients	parameter	coefficients
age	n.s.	age	n.s.	age	n.s.
gender	n.s.	gender	n.s.	gender	n.s.
AL	n.s.	AL	n.s.	**AL**	**0.54**
**CCT**	0.022	CCT	n.s.	CCT	n.s.
**average corneal curvature**	-2.5	average corneal curvature	n.s.	**average corneal curvature**	**-4.4**
		**A time-1**	**11.6**		
		A length-1	n.s.	A length-1	n.s.
		**A velocity-1**	**35.4**	A velocity-1	n.s.
		A time-2	n.s.	A time-2	n.s.
		A length-2	n.s.	A length-2	n.s.
		A velocity-2	n.s.	**A velocity-2**	**10.1**
		highest concavity time	n.s.	**highest concavity time**	**2.4**
		**highest concavity curvature**	**0.67**	highest concavity curvature	n.s.
		peak distance	n.s.	peak distance	n.s.
		maximum deformation amplitude	n.s.	**maximum deformation amplitude**	**-23.8**

## Discussion

In the current study, the relationship between CST parameters and CCT, corneal curvature, AL, age and IOP was investigated. As a result, it was suggested that six out of ten CST parameters are influenced by IOP; namely: *A time-1*, *A velocity-1*, *A time-2*, *A velocity-2*, *highest concavity curvature*, and *maximum deformation amplitude*. AL was a significant predictor of *A time-1*, *A velocity-1*, *A time-2*, *A velocity-2*, *highest concavity time*, *highest concavity curvature*, and also *maximum deformation amplitude*. CCT was a significant predictor of *A length-2*, *A velocity-2*, and also *highest concavity curvature*. Average corneal curvature was selected in a model for *A time-1*, *A length-1*, *A velocity-2*, and *highest concave curvature*. In addition, it was suggested that IOP measured with Goldmann tonometer is influenced by CST determined-CCT and corneal curvature when other CST parameters were not considered, but importantly, these parameters were no longer selected when the other CST parameters were considered. Instead, it was suggested that *A time-1*, *A velocity-1* and *highest concave curvature* were significantly related to IOP-G. The mechanism of *A time-1* measurement is identical to that in non-contact tonometry; the time to applanation is measured following an air-puff injection where force increases with time.[34] Interestingly, as shown in the Table 5, totally different parameters were identified when *A time-1* was included or excluded. These excluded/included parameters are closely inter-correlated and further study is needed to identify the reason of the exclusion/inclusion of the parameters, in particular shedding light on the inter-correlation of the parameters. However, most importantly, even in the absence of *A time-1 as an explanatory variable*, CCT was not selected in a model for IOP-G. In a previous study[35], it is reported that CCT is related to *A time-1* and also *maximum deformation amplitude*, in contradiction with our current result (Table 4). This is probably because the effect of CCT was masked by (wrapped in) IOP-G which was not included in the analysis in the previous report. The effect of CCT on IOP-G could be observed if CCT with larger variation is included, such as post-LASIK eye. A further study should be carried out to shed light on this issue.

Interestingly, IOP-G was selected in the optimal linear model for *A time-1* (positive coefficient), *A velocity-1* (negative coefficient), and inversely, *A time-2* (negative coefficient), and also *A velocity-2* (positive coefficient, but IOP-G was not selected in the model for *A length-1 and -2*. This implies that IOP level does not change the length of the first (inward) and second (outward) applanations, but the inward applanation occurs late and slowly with high IOP, and outward applanation occurs fast and quickly with high IOP.

These findings suggest measured CST parameter values are dependent on the level of IOP. Thus, it is recommended that these parameters be corrected for IOP; otherwise the magnitude of the air-puff jet could be adjusted for IOP, which is the method conducted in ORA.[36] Some CST parameters were not affected by IOP but it could be argued that these particular parameters do not reflect corneal hysteresis. A further study should be carried out to investigate if CST parameters are useful for analyzing the progression of glaucoma, in conjunction with ORA-derived measurements of CH. shown in Table 4, *highest corneal curvature* increases with increasing IOP-G, increasing CCT, and increasing corneal curvature; it was also related to AL and age. In addition, this values showed low CV and also relatively high ICC values. This hints of the possible usefulness of this parameter for evaluating biometric properties of cornea. Interestingly, *A time-2* and *maximum deformation amplitude* showed low CV and high ICC values, and these parameters were related with IOP-G, age and AL, but not with corneal biomechanical parameters of CCT and average corneal curvature.

The viscoelastic aspect of the cornea is represented by ‘hysteresis’. Hysteresis is a measure of the energy absorption during the ‘loading/unloading’ stress/strain cycle of viscoelastic materials[37] and as a result, is related to IOP measurements using the Goldmann applanation tonometer.[20] To date, previous studies have largely investigated corneal hysteresis using ORA;[38–48] CST parameters may be reflecting other aspects of corneal biomechanics and this study represents the first report to have investigated the influence of CST corneal parameters, on IOP measured with Goldmann applanation tonometry, although there is a previous report which compared IOP measured with CST and IOP-G.[49]In agreement with previous studies,[9–19] CCT was selected in the optimal model for IOP-G with a positive coefficient, when CST parameters other than CCT were not analyzed. Similarly, average corneal curvature was also included, agreeing with previous reports.[13,20,21] However, these parameters were no longer selected in the optimal model for IOP-G when various CST parameters were included; instead *A time-1*, *A velocity-1* and also *highest concavity curvature* were selected. As shown in Table 4, *highest concavity curvature* is related to CCT and average corneal curvature, and hence inclusion of this parameter could have resulted in the exclusion of CCT and average corneal curvature. Thus, the current results suggest that CST parameters are more influential to IOP-G than CCT and average corneal curvature. However, as shown in Table 4, *A time-1* and *A velocity-1* are changeable according to the level of IOP, hence, in order to validate this finding, a future experiment is needed in which parameters are corrected for IOP or the force of the air-puff is adjusted for IOP so that CST parameters are independent of IOP.

A possible caveat of the current study is the cross sectional design; a further study should be carried out to validate the results associated with age. In addition, the influence of changing IOP, such as with diurnal[39], inter-date or pharmacological change,[50] should be carried out to confirm the current results showing an association between IOP and CST parameters. For example, a flattened cornea will return to the original shape quickly, and only a small area will be flattened when IOP is high. Indeed, in ORA, the magnitude of the pressure given to flatten cornea is adjusted by IOP, because ORA measured CH is largely affected by IOP[50], whereas this adjustment is not performed in CST. It was shown that CV and ICC varied among the different CST parameters (see Table 3). However, in general, this variance was independent of the particular recording, which suggests this variance occurred in random. Thus, taking the average value of repeated CST-measurements appears a valid approach, especially for *A length-1*, *A velocity-1*, *A-length-2*, and *peak distance*. A previous study suggested similar tendency of these parameters as a result of two times repeated measurements[5], however the current study suggested CV and ICC were not sufficient for these parameters even with three repeats. In addition, because of these issues, same series of optimal linear model selection was carried out using M-estimator robust regression, instead of simple linear regression, which is robust to outliers.[51] As a result, although the coefficients were very slightly different, completely same parameters were selected in all optimal models, except for one exception: AL was not selected for A velocity-1 (data not shown in Result).

In conclusion, this study suggests that CST parameters are not closely related to CCT, but are significantly affected by IOP. Our results also demonstrated that IOP-G was more closely related to CST-parameters than CCT and average corneal curvature, however, this result should be validated in a future study in which CST parameters are corrected for IOP level.

## Supporting Information

S1 File Data analyzed. (CSV)
